# Utility of including BRAF mutation analysis with ultrasonographic and cytological diagnoses in ultrasonography-guided fine-needle aspiration of thyroid nodules

**DOI:** 10.1371/journal.pone.0202687

**Published:** 2018-08-17

**Authors:** Da Som Kim, Dong Wook Kim, Young Jin Heo, Jin Wook Baek, Yoo Jin Lee, Hye Jung Choo, Young Mi Park, Ha Kyoung Park, Tae Kwun Ha, Do Hun Kim, Soo Jin Jung, Ji Sun Park, Ki Jung Ahn, Hye Jin Baek, Taewoo Kang

**Affiliations:** 1 Department of Radiology, Busan Paik Hospital, Inje University College of Medicine, Busan, South Korea; 2 Department of General Surgery, Busan Paik Hospital, Inje University College of Medicine, Busan, South Korea; 3 Department of Otorhinolaryngology-Head and Neck Surgery, Busan Paik Hospital, Inje University College of Medicine, Busan, South Korea; 4 Department of Pathology, Busan Paik Hospital, Inje University College of Medicine, Busan, South Korea; 5 Department of Nuclear Medicine, Busan Paik Hospital, Inje University College of Medicine, Busan, South Korea; 6 Department of Radiation Oncology, Busan Paik Hospital, Inje University College of Medicine, Busan, South Korea; 7 Department of Radiology, Gyeongsang National University Changwon Hospital, Gyeongsang National University School of Medicine, Changwon, South Korea; 8 Department of Surgery (Busan Cancer Center), Pusan National University Hospital, Pusan National University College of Medicine, Busan, South Korea; 2nd medical school of Charles University, CZECH REPUBLIC

## Abstract

This study investigated the role of BRAF mutation analysis in thyroid fine-needle aspiration (FNA) samples compared to ultrasonographic and cytological diagnoses. A total 316 patients underwent ultrasonography (US)-guided FNA with BRAF^V600E^ mutation analysis to diagnose thyroid nodules. One hundred sixteen patients with insufficient US images (n = 6), follow-up loss (n = 43), or unknown final diagnosis (n = 67) were excluded from the study. Comparisons between US diagnoses, cytological diagnoses, and BRAF mutation analysis were performed. Of 200 thyroid nodules, there was US diagnosis with 1 false negative and 11 false positive cases, cytological diagnosis with 10 false negative and 2 false positive cases, and BRAF^V600E^ mutation analysis with 19 false negative and 2 false positive cases. The sensitivity, specificity, positive and negative predictive values, and accuracy of BRAF^V600E^ mutation analysis were 83.2%, 98.1%, 97.5%, 86.6%, and 91%, respectively. Of the 18 nodules with Bethesda category III, 9 were true positive, 6 were true negative, 3 was a false negative, and none were false positive on BRAF mutation analysis. In conclusion, we recommend that BRAF^V600E^ mutation analysis only be performed for evaluating thyroid nodules with Bethesda category III, regardless of US diagnosis.

## Introduction

Ultrasonography (US)-guided fine-needle-aspiration (FNA) is a simple and accurate tool for evaluating thyroid nodules [1]. With the rising incidence of thyroid cancer and improved nodule detection methods, more thyroid nodules are evaluated by US-guided FNA and cytology. However, these techniques have several limitations, such as operator dependency, different results according to nodular composition and size, false negative or positive cytological findings, and indeterminate cytology [1]. In cases of indeterminate cytology, there is a need for ancillary tests that may be used as adjuncts to thyroid cytology so that the additional information can be used to treat patients more effectively [2]. Genetic alterations have been shown to play a pathogenetic role in thyroid tumorigenesis, and several molecular markers have been investigated for their applicability to thyroid FNA [3, 4]. Among the several Raf kinase isoforms, the B type Raf kinase (BRAF) is the strongest activator of the downstream mitogen-activated extra-cellular signal regulated kinase signaling pathway [3, 4]. BRAF^V600E^ mutations are associated with early tumorigenesis and aggressive behavior of papillary thyroid carcinoma (PTC), and are considered a specific marker of PTC [5, 6].

BRAF^V600E^ is the most common type of BRAF mutation, and has been used as an adjunctive diagnostic tool for patients with thyroid nodules [7]. Several studies have shown that including BRAF mutation analysis with FNA significantly improves the diagnostic accuracy [7–9]. In particular, BRAF mutation analysis can be helpful for the diagnosis of thyroid nodules with atypia of undetermined significance on cytology [2, 9–11]. Additionally, BRAF^V600E^ mutation analysis has a high positive predictive value (95.5–100.0%) for the diagnosis of malignancies in thyroid nodules with atypia of undetermined significance [2, 9–11]. Nevertheless, whether BRAF^V600E^ analysis should be routinely used in clinical practice remains controversial. Numerous researchers have shown BRAF^V600E^ mutation analysis to be an effective diagnostic approach for thyroid FNA samples, while others believe that its utility is limited owing to the low prevalence of BRAF^V600E^ mutations in indeterminate nodules [2, 9–11]. In this study, we assessed the diagnostic role and characteristics of BRAF^V600E^ mutation analysis in thyroid FNA compared to US diagnosis and cytology.

## Materials and methods

### Study population

**Fig 1:**
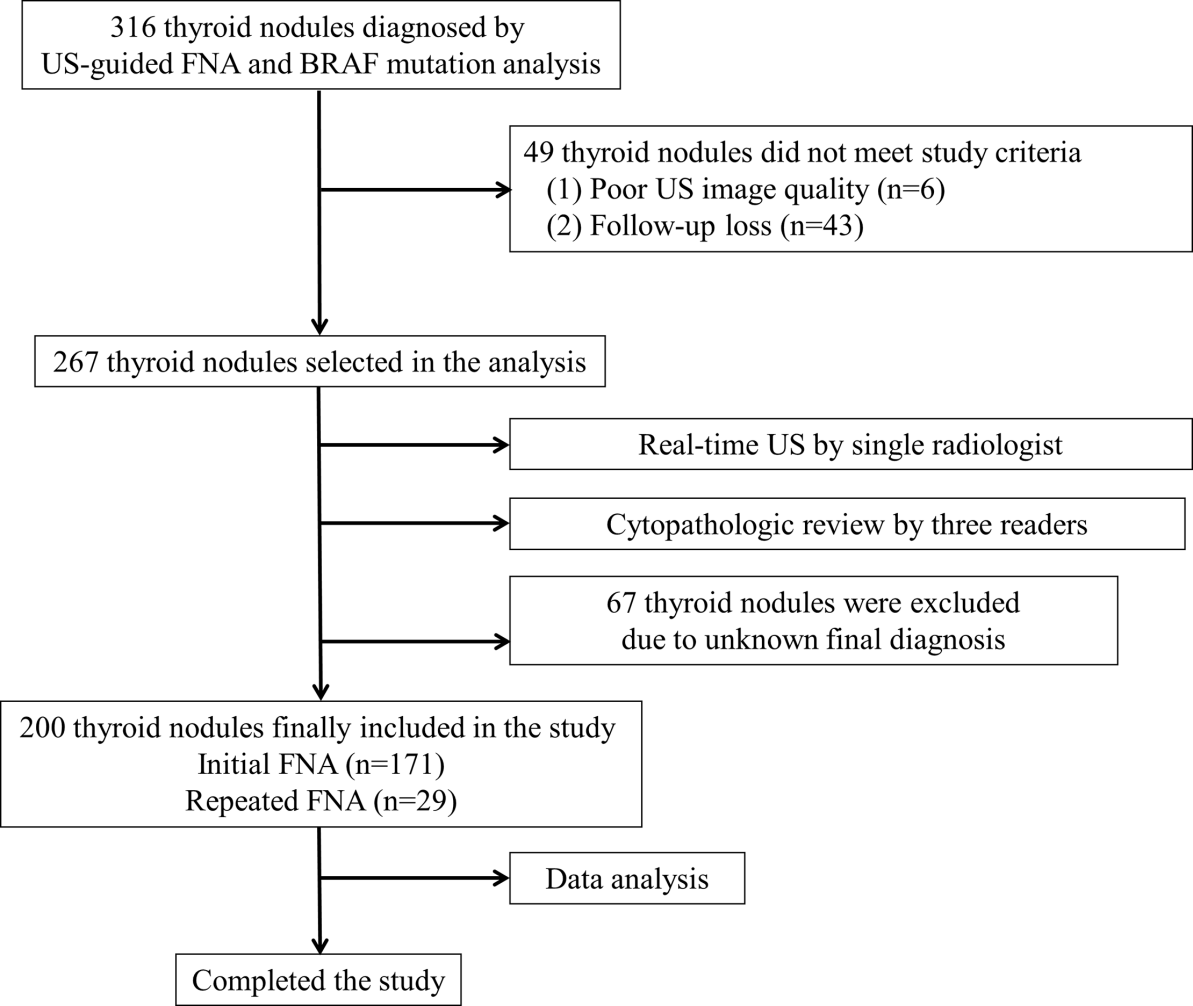
Diagram illustrating the enrollment of subjects using the study’s algorithm.

This retrospective study was approved by the Busan Paik Hospital institutional review board (IRB 2016-10-0168); the requirement for informed consent was waived. A single radiologist (who had performed >500 US-guided FNAs/year over 11 years) conducted US-guided FNA to diagnose 316 thyroid nodules (mean size, 12.9 ± 10.1 mm; range, 2.9–52.1 mm) in 316 patients (258 women, 58 men; mean age, 49.8 years; age range, 9–86 years) between January 2013 and December 2015. Among them, there were 146 subcentimetric thyroid nodules. The reasons of US-FNA for these subcentimetric nodules included suspicious US features (n = 95), the presence of contralateral thyroid malignancy (n = 8), and patients’ requests (n = 43). Of the 316 thyroid nodules, 6 nodules with unclear US images and 43 that were lost to follow-up were excluded from the study (Fig 1).

### Thyroid US and nodule classification

Real-time thyroid US was performed by the same radiologist, using a high-resolution US instrument (iU 22; Philips Medical Systems, Bothell, WA, USA) equipped with a 12–15 MHz linear probe. For thyroid nodules, benign US features included an ovoid shape, isoechogenicity, a smooth margin, and peripheral vascularity; borderline US features of thyroid nodules included hypoechogenicity, centrally predominant vascularity, and a solid, round configuration; and malignant US features included marked hypoechogenicity, a spiculated margin, microcalcifications, and a taller-than-wide shape [12, 13]. US diagnosis for each thyroid nodule was classified into 1 of 5 categories based on the real-time US examination results: “benign” (3 or more benign US features and no malignant or borderline US features), “probably benign” (1 or 2 benign US features and no malignant or borderline US features), “indeterminate” (1 or more borderline US features and no malignant US features), “suspicious for malignancy” (1 malignant US feature, regardless of benign or borderline US features), or “malignant” (2 or more malignant US features) [12, 13]. In the calculation of diagnostic accuracy in US diagnosis, “benign” and “probably benign” categories were classified as benign, whereas “suspicious for malignancy” and “malignant” categories were classified as malignancy, but “indeterminate” US category cases were excluded.

### US-guided FNA and cytological diagnosis

US-guided FNA was performed immediately after thyroid US by the same radiologist using a 1-needle puncture, no aspiration device, and no local anesthesia [14]. For each sample, a smear was prepared on 4–6 slides using the flipping-extraction technique; samples were fixed in 95% ethanol and examined after Papanicolaou staining. The cytological analysis was categorized as follows: inadequate (Bethesda category I), benign (Bethesda category II), atypia of undetermined significance or follicular lesion of undetermined significance (Bethesda category III), suspicious for a follicular neoplasm (Bethesda category IV), suspicious for malignancy (Bethesda category V), or malignant (Bethesda category VI) [15]. Three cytopathologists with different levels of experience (13, 14, and 20 years) who were blinded to the US-based diagnoses performed the cytological diagnoses. In the calculation of diagnostic accuracy in cytological diagnosis, Bethesda category I and II were classified as benign, whereas Bethesda category V and VI were classified as malignancy, but “indeterminate” cytology cases (Bethesda category III and IV) were excluded.

### DNA extraction and BRAF^V600E^ mutation analysis

After US-guided FNA for cytology, aspiration was repeated for BRAF^V600E^ mutation analysis. Genomic DNA was extracted from obtained aspirates using the QIAamp DNA extraction kit (Qiagen, Hilden, Germany) according to the manufacturer’s protocol. T1799A transversion was detected using the Anyplex BRAF^V600E^ real-time detection system (Seegene Inc., Seoul, Korea). Genomic DNA (5 μL) was added to the 15 μL reaction mixture (2 μL of 10× BRAF Oligo Mix, 3 μL of 8-methoxypsoralen solution, and 10 μL of 2× Anyplex PCR Master Mix [Seegene Inc., Seoul, Korea]). Real-time polymerase chain reaction was performed using a CFX96 real-time polymerase chain reaction system (Bio-Rad, Hercules, CA, USA) with the following conditions: 15 min at 95°C, followed by 15 cycles of 15 s at 95°C and 30 s at 60°C, and then by 35 cycles of 30 s at 95°C and 32 s at 60°C. For real-time polymerase chain reaction, the cycle threshold was defined as the number of cycles required for the fluorescent signal to exceed the threshold. The cycle threshold values of the target and internal control were <33 and 30, respectively. Each run contained both a positive and negative control. In the calculation of diagnostic accuracy in BRAF mutation analysis, negatives were classified as benign, whereas positives were classified as malignancy.

### Final diagnosis

In our hospital, thyroid surgery is performed as follows: (1) malignant cytology (Bethesda category V and VI), (2) positive BRAF mutation analysis, (3) indeterminate cytology (Bethesda category III and IV) but suspicious US diagnosis (“suspicious for malignancy” and “malignant” categories), and (4) patient request. On the other hand, patients with nodules that were diagnosed as benign on both US and cytology received follow-up by thyroid US instead of undergoing thyroid surgery or repeated US-guided FNA. The final diagnoses of the thyroid nodules were determined as follows: (1) when surgery was performed, diagnosis was according to the histopathological results; (2) when nonsurgical thyroid nodules in the “benign” or “probably benign” US categories exhibited benign cytological results and no suspicious features on follow-up US, they were considered benign; and (3) when nonsurgical thyroid nodules in the “indeterminate,” “suspicious for malignancy,” or “malignant” US categories exhibited benign cytological results on both initial and repeated US-guided FNAs, they were considered benign.

### Statistical analysis

The data were tested for normal distribution by employing the Kolmogorov-Smirnov test. The age at the time of diagnosis and sizes of thyroid nodules are expressed as means ± standard deviations. Mean differences in age and sizes of thyroid nodules between the 2 groups (BRAF mutation-positive vs. BRAF mutation-negative) were compared using the independent t-test. Group comparisons of categorical variables related to US diagnoses, FNA results, and final diagnoses were performed using the χ^2^ test or, for small cell values, the Fisher’s exact test. The diagnostic indices (sensitivity, specificity, positive and negative predictive values, and accuracy) of BRAF mutation analyses were also calculated. All statistical analyses were performed using the IBM SPSS Statistics 19.0 software package (SPSS, Chicago, IL, USA). A P-value <0.05 was considered statistically significant.

## Results

Of the 267 thyroid nodules (mean diameter, 12.8 ± 9.7 mm; range, 2.9–52.1 mm) in 267 patients (223 women, 44 men; mean age, 52.1 ± 12.1 years; age range, 18–84 years), 67 (mean diameter, 9.8 ± 6.7 mm; range, 2.9–37.6 mm) were excluded because of an unknown final diagnosis (Fig 1). Ultimately, 200 thyroid nodules (mean diameter, 13.8 ± 10.3 mm; range, 3.0–52.1 mm) in 200 patients (167 women, 33 men; mean age, 51.5 ± 12.7 years; age range, 18–79 years) were included, representing 171 (85.5%) initial and 29 (24.5%) repeated FNAs. The locations of the thyroid nodules included the right thyroid lobe (n = 108), left thyroid lobe (n = 85), and isthmus (n = 7). The final diagnoses of the 200 thyroid nodules were determined as follows: (1) histopathological analysis (n = 106); (2) benign diagnoses according to US and cytology, with no US follow-up (n = 59); and (3) indeterminate or malignant on US, but benign cytological results based on both initial and repeated US-guided FNAs (n = 35). There were 95 PTCs, 1 follicular thyroid carcinoma, 2 medullary thyroid carcinomas, 3 follicular adenomas, 5 nodular hyperplasia, and 94 non-surgical benign nodules. The nodule size was significantly larger in the BRAF mutation-negative group (14.8 mm) than in the BRAF mutation-positive group (9.1 mm) (p < 0.0001), whereas there was no significant difference in patient age (p = 0.146).

US and cytological diagnoses as well as BRAF^V600E^ mutation analyses of the 200 thyroid nodules are summarized in Table 1, along with their final diagnostic results. Of the benign US group (n = 82), 2 benign thyroid nodules were positive on BRAF mutation analysis, whereas they showed Bethesda category II on cytology. In the indeterminate US group (n = 20), no nodules were positive on BRAF mutation analysis. Two malignant thyroid nodules with indeterminate US diagnoses and negative BRAF mutation analysis were diagnosed as PTC and follicular thyroid carcinoma after thyroid surgery, respectively. In the malignant US group (n = 98), 17 malignant nodules were negative on BRAF mutation analysis (15 PTCs and 2 medullary thyroid carcinomas). Among these cases, 11 showed malignant cytology, the remainders had Bethesda category I (n = 1), II (n = 2), and III (n = 3). In the benign cytology group (Bethesda category I or II, n = 104), 4 malignant nodules were negative on BRAF mutation analysis (3 PTCs and 1 follicular thyroid carcinoma) and 2 benign nodules were positive on BRAF mutation analysis (1 nodular hyperplasia and 1 non-surgical benign nodule). In the indeterminate cytology group (n = 18), 12 nodules were surgically removed because of positive BRAF mutation analysis (n = 9) and malignant US diagnosis (n = 3). In the malignant cytology group (Bethesda category V or VI, n = 78), there were 12 malignant nodules with negative BRAF mutation analysis (10 PTCs and 2 medullary thyroid carcinomas). As for the diagnostic indices of BRAF mutation analyses for the 200 thyroid nodules, BRAF^V600E^ mutation analysis revealed 79 true positive, 2 false positive, 103 true negative, and 16 false negative cases; the sensitivity, specificity, positive and negative predictive values, and accuracy were 83.2%, 98.1%, 97.5%, 86.6%, and 91%, respectively. When medullary and follicular thyroid carcinoma cases were excluded, the sensitivity, specificity, positive and negative predictive values, and accuracy of BRAF mutation analysis were 83.2%, 98.0%, 97.5%, 86.2%, and 90.9%, respectively.

**Table 1:** Ultrasonographic diagnoses, cytological diagnoses, and BRAF mutation analyses of 200 thyroid nodules according to final results.

	BRAF mutation analysis	
	Negative (n = 119, 59.5%)	Positive (n = 81, 40.5%)
**US diagnosis**		
Benign (n = 44, 22%)	NH (1), NSBN (41); (n = 42, 21%)	NH (1), NSBN (1); (n = 2, 1%)
Probably benign (n = 38, 19%)	NH (2), FA (2), NSBN (33); (n = 37, 18.5%)	PTC (1); (n = 1, 0.5%)
Indeterminate (n = 20, 10%)	PTC (1), FTC (1), FA (1), NSBN (9); (n = 12, 6%)	PTC (8); (n = 8, 4%)
Suspicious for malignancy (n = 57, 28.5%)	PTC (10), MTC (1), NH (1), NSBN (10); (n = 22, 11%)	PTC (35); (n = 35, 17.5%)
Malignant (n = 41, 20.5%)	PTC (5), MTC (1); (n = 6, 3%)	PTC (35); (n = 35, 17.5%)
**Cytological diagnosis** **(Bethesda category)**		
I (n = 5, 2.5%)	PTC (1), NSBN (3); (n = 4, 2%)	PTC (1); (n = 1, 0.5%)
II (n = 99, 49.5%)	PTC (2), FTC (1), FA (3), NH (2), NSBN (84); (n = 92, 46%)	PTC (5), NH (1), NSBN (1); (n = 7, 3.5%)
III (n = 18, 9%)	PTC (3), NSBN (6); (n = 9, 4.5%)	PTC (9); (n = 9, 4.5%)
IV (n = 0, 0%)	0	0
V (n = 25, 12.5%)	PTC (3), NH (2); (n = 5, 2.5%)	PTC (20); (n = 20, 10%)
VI (n = 53, 26.5%)	PTC (7), MTC (2); (n = 9, 4.5%)	PTC (44); (n = 44, 22%)

Note.—Numbers in parentheses are prevalence of each item. US, ultrasonography; NH, nodular hyperplasia; NSBN, Non-surgical benign nodules, which were finally determined by repeated fine-needle aspiration cytology or fine-needle aspiration cytology and follow-up ultrasonographic findings. FA, follicular adenoma; PTC, papillary thyroid carcinoma; FTC, follicular thyroid carcinoma; MTC, medullary thyroid carcinoma.

Of the 200 thyroid nodules, 171 (mean diameter, 14.2 ± 10.4 mm; range, 3.0–52.1 mm) had no previous FNA history; the US diagnoses, cytological diagnoses, and BRAF^V600E^ mutation analyses of these thyroid nodules that underwent initial US-guided FNA are summarized in Table 2 according to their final results. In the benign US diagnosis group (n = 69), 2 nodules (2.9%) were positive on BRAF mutation analysis, whereas they were Bethesda category II on cytology. In the malignant US diagnosis group (n = 86), 13 nodules were false negative on BRAF mutation analysis (13 PTCs). Among these false negative cases, 10 showed malignant cytology, whereas the remainders were Bethesda category II (n = 2) and III (n = 2). In the benign cytology group (n = 87), 2 benign nodules (1 nodular hyperplasia and 1 non-surgical benign nodule) were false positive and 3 malignant nodules (2 PTCs and 1 follicular thyroid carcinoma) were false negative on BRAF mutation analysis. In the malignant cytology group (n = 70), 10 malignant nodules (10 PTCs) were false negative on BRAF mutation analysis.

**Table 2:** Ultrasonographic diagnoses, cytological diagnoses, and BRAF mutation analyses of 171 thyroid nodules with the initial fine-needle aspiration according to final results.

	BRAF mutation analysis	
	Negative (n = 95, 55.6%)	Positive (n = 76, 44.4%)
**US diagnosis**		
Benign (n = 39, 22.8%)	NSBN (37); (n = 37, 21.6%)	NH (1), NSBN (1); (n = 2, 1.2%)
Probably benign (n = 30, 17.5%)	FA (1), NH (1), NSBN (27); (n = 29, 17.0%)	PTC (1); (n = 1, 0.6%)
Indeterminate (n = 16, 9.4%)	PTC (1), FTC (1), FA (1), NSBN (6); (n = 9, 3.5%)	PTC (7); (n = 7, 4.1%)
Suspicious for malignancy (n = 50, 29.2%)	PTC (10), NH (1), NSBN (5); (n = 16, 9.4%)	PTC (34); (n = 34, 19.9%)
Malignant (n = 36, 21.1%)	PTC (3), MTC (1); (n = 4, 2.3%)	PTC (32); (n = 32, 18.7%)
**Cytological diagnosis (Bethesda category)**		
I (n = 3, 1.8%)	NSBN (2); (n = 2, 1.2%)	PTC (1); (n = 1, 0.6%)
II (n = 84, 49.1%)	PTC (2), FTC (1), FA (2), NH (2), NSBN (70); (n = 77, 45.0%)	PTC (5), NH (1), NSBN (1); (n = 7, 4.1%)
III (n = 14, 8.2%)	PTC (2), NSBN (3); (n = 5, 2.9%)	PTC (9); (n = 9, 3.5%)
IV (n = 0, 0%)	0	0
V (n = 21, 12.3%)	PTC (3); (n = 3, 1.8%)	PTC (18); (n = 18, 10.5%)
VI (n = 49, 28.7%)	PTC (7), MTC (1); (n = 8, %)	PTC (41); (n = 41, 24.0%)

Note.—Numbers in parentheses are prevalence of each item. US, ultrasonography; NSBN, Non-surgical benign nodules, which were finally determined by repeated fine-needle aspiration cytology or fine-needle aspiration cytology and follow-up ultrasonographic findings. NH, nodular hyperplasia; FA, follicular adenoma; PTC, papillary thyroid carcinoma; FTC, follicular thyroid carcinoma; MTC, medullary thyroid carcinoma.

The 29 thyroid nodules that had undergone two or more US-guided FNA sessions (mean diameter, 11.8 ± 9.7 mm; range, 4.1–48.0 mm) are summarized in Table 3. Among them, 15 nodules (51.7%) had Bethesda category III on cytology after the initial US-guided FNA, including 4 PTCs, 1 follicular adenoma, 1 nodular hyperplasia, and 9 non-surgical benign nodules. Of the 4 PTCs, 3 were BRAF^V600E^-mutation positive and 1 was mutation-negative. No false positive cases were found in this group following BRAF mutation analysis.

**Table 3:** Ultrasonographic diagnoses, cytological diagnoses, and BRAF mutation analyses of 29 thyroid nodules with the repeated fine-needle aspiration according to final results.

	BRAF mutation analysis	
	Negative (n = 24)	Positive (n = 5)
**US diagnosis**		
Benign (n = 5)	NH (1), NSBN (4)	0
Probably benign (n = 8)	FA (1), NH (1), NSBN (6)	0
Indeterminate (n = 4)	NSBN (3)	PTC (1)
Suspicious for malignancy (n = 7)	MTC (1), NSBN (5)	PTC (1)
Malignant (n = 5)	PTC (2)	PTC (3)
**Cytological diagnosis** **(Bethesda category)**		
I (n = 2)	PTC (1), NSBN (1)	0
II (n = 15)	FA (1), NSBN (14)	0
III (n = 4)	PTC (1), NSBN (3)	0
IV (n = 0)	0	0
V (n = 4)	NH (2)	PTC (2)
VI (n = 4)	MTC (1)	PTC (3)

Note.—Numbers in parentheses are prevalence of each item. US, ultrasonography; NH, nodular hyperplasia; NSBN, Non-surgical benign nodules, which were finally determined by repeated fine-needle aspiration cytology or fine-needle aspiration cytology and follow-up ultrasonographic findings. FA, follicular adenoma; MTC, medullary thyroid carcinoma; PTC, papillary thyroid carcinoma; FTC, follicular thyroid carcinoma.

## Discussion

BRAF mutation analysis has been used to improve the diagnostic accuracy in thyroid FNA. However, the rate of BRAF mutation positivity ranges from 29% to 69% in previous studies [6, 9, 15]. To date, the routine application of BRAF mutation analysis is not recommended because of its cost and questionable diagnostic utility [6]. In the literature, BRAF mutation analysis is recommended on thyroid nodules with indeterminate cytology [6, 10]. Our results concurred with this guideline. Of the 15 repeated-FNA nodules that were Bethesda category III in the initial US-guided FNA, 3 PTCs were surgically excised because of BRAF^V600E^ mutation positivity.

US diagnosis is considered useful for evaluating thyroid nodules [1, 12]. Moreover, US-guided FNA has been used for the diagnosis of thyroid nodules with suspicious US features [1, 13]. Thus, both US diagnosis and US-guided FNA appear to be sufficient for the evaluation and management of thyroid nodules. However, these methods are unhelpful for thyroid nodules with malignant US features and benign cytology or for thyroid nodules with indeterminate cytology [1, 15, 16]. Hence, BRAF^V600E^ mutation analysis was introduced, which several studies found to be more effective in thyroid nodules with suspicious US features than in those with benign US features [17–19]. In the malignant US group in our study, BRAF^V600E^ mutation analysis exhibited no false positives, whereas false negatives on BRAF mutation analysis were found in 15 PTCs and 2 medullary thyroid carcinomas. Of the benign US group (n = 82), 4 nodules were false positive on BRAF^V600E^ mutation analysis (showing benign cytology), whereas there were no false negative cases. Thus, the routine application of BRAF^V600E^ mutation analysis is not recommended in thyroid FNA.

In case of discordance between the US and cytological diagnoses of thyroid nodules, cytological diagnoses take priority [20]. However, in the management of thyroid nodules that have benign cytology but are positive on BRAF mutation analysis, thyroidectomy should be considered in nodules that have 2 or more malignant US features [21]. In our study, 2 false positive BRAF^V600E^ mutation cases were benign on US and cytology, while 16 false negative BRAF^V600E^ mutation cases were malignant on US or cytology. Thus, a combination of US diagnosis, cytological diagnoses, and BRAF^V600E^ mutation analysis may be required for a reliable diagnosis.

BRAF mutation analysis may be effective for the initial US-guided FNA of thyroid nodules with suspicious US features [18]. However, the routine use of BRAF mutation analysis may not be cost-effective in clinical practice. In our study, 85.5% of the nodules (171/200) had undergone initial FNA. In the benign US diagnosis group (n = 69), there were 2 false positive cases on BRAF^V600E^ mutation analysis; these cases showed benign cytology. In the benign cytology group (n = 87), there were 2 false positive cases on BRAF^V600E^ mutation analysis. Such false positive results may prevent patients from receiving the appropriate treatment; therefore, the routine use of BRAF^V600E^ mutation analysis at the time of the initial FNA of the thyroid nodules is unlikely to be helpful.

There are several limitations to this study. First, not all thyroid nodules were surgically confirmed. Ninety-five non-surgical benign nodules with only repeated US-guided FNA or US follow-up were included. Second, we excluded 67 non-surgical thyroid nodules from the calculation of the diagnostic index; this may have resulted in selection bias. Third, of the 200 thyroid nodules investigated, there were no cases with Bethesda category IV. Fourth, the PTC subtypes were not evaluated. Finally, three cytopathologists with different levels of experience in cytological analysis interpreted the FNA slides; however, we did not investigate interobserver variability.

## Conclusions

BRAF^V600E^ mutation analysis is not particularly helpful in cases of benign US diagnosis. Furthermore, BRAF^V600E^ mutation analysis at the time of the initial FNA of thyroid nodules may not be of benefit because of the lack of cost-effectiveness in clinical practice. Therefore, we suggest that BRAF^V600E^ mutation analysis should be performed only for evaluating thyroid nodules with Bethesda category III, regardless of US diagnosis.
